# Covid -19 pandemic‘s impact on the clinical presentation of brief psychotic disorders

**DOI:** 10.1192/j.eurpsy.2022.1376

**Published:** 2022-09-01

**Authors:** S. Khouadja, C. Najjar, Y. Brigui, A. Arbi, S. Younes, L. Zarrouk

**Affiliations:** University Hospital of Mahdia, Tunisia, Psychiatry, Mahdia, Tunisia

**Keywords:** brief psychotic disorder, Covid-19, delusions, stressors

## Abstract

**Introduction:**

A major increase in mental health issues was noted since the outbreak of the covid-19 pandemic even in patients with no history of mental health illness, specifically brief psychotic disorders.

**Objectives:**

Establish the covid-19 pandemic circumstances as precipitating factors of psychosis independently from other stressors

**Methods:**

This is a cross-sectional and descriptive study carried out in the psychiatric department of the University Hospital of Mahdia including two groups of patients over a period of 15 months: From October 2018 to December 2019 are pre-covid cases, and the second group consists of the ones admitted between June 2020 and August 2021. We have collected the data of patients diagnosed with brief psychotic disorder according to DSM-5. We have focused on two clinical characteristics of the psychotic episodes (theme of delusions, stressors).

**Results:**

We have collected 19 patients, 12 among them during the pre-covid period. In our pre-covid period, brief psychotic disorder presented mostly with marked stressors (33.3%) whilst in the middle of the pandemic, marked stressors are present in only 14.3% of the cases. Which could suggest that this state of alarm can singlehandedly trigger psychosis. For the theme of delusions, in the pre-covid period, the religious theme appeared to be the most frequent (58.3%), whilst during the pandemic, persecution became the most prevalent (71.4%), showing how living in fear of contracting the virus could manifest itself in delusional content.

**Conclusions:**

Living in a prolonged state of alarm is, in itself, a marked stressor, theoretically capable of increasing the psychosis rate and altering its characteristics.

**Disclosure:**

No significant relationships.

